# A glycine zipper motif mediates the formation of toxic β-amyloid oligomers *in vitro *and *in vivo*

**DOI:** 10.1186/1750-1326-6-61

**Published:** 2011-08-23

**Authors:** Virginia Fonte, Vishantie Dostal, Christine M Roberts, Patrick Gonzales, Pascale Lacor, Jordi Magrane, Natalie Dingwell, Emily Y Fan, Michael A Silverman, Gretchen H Stein, Christopher D Link

**Affiliations:** 1 Institute for Behavioral Genetics, University of Colorado, Boulder, CO 80309, USA; 2 Neurobiology and Physiology Department, Northwestern University, Evanston, IL, 60208, USA; 3 Department of Neurology and Neuroscience, Weill Medical College of Cornell University, New York, NY 10065, USA; 4 Department of Biological Sciences, Simon Fraser University, Burnaby, British Columbia V5A 1S6, Canada; 5 Department of Molecular, Cellular, and Developmental Biology, University of Colorado, Boulder, CO 80309, USA; 6 Integrative Physiology, University of Colorado, Boulder, CO 80309, USA

**Keywords:** Alzheimer's disease, *C. elegans*, pore-forming toxin, glycine motif

## Abstract

**Background:**

The β-amyloid peptide (Aβ) contains a Gly-XXX-Gly-XXX-Gly motif in its C-terminal region that has been proposed to form a "glycine zipper" that drives the formation of toxic Aβ oligomers. We have tested this hypothesis by examining the toxicity of Aβ variants containing substitutions in this motif using a neuronal cell line, primary neurons, and a transgenic *C. elegans *model.

**Results:**

We found that a Gly37Leu substitution dramatically reduced Aβ toxicity in all models tested, as measured by cell dysfunction, cell death, synaptic alteration, or tau phosphorylation. We also demonstrated in multiple models that Aβ Gly37Leu is actually anti-toxic, thereby supporting the hypothesis that interference with glycine zipper formation blocks assembly of toxic Aβ oligomers. To test this model rigorously, we engineered second site substitutions in Aβ predicted by the glycine zipper model to compensate for the Gly37Leu substitution and expressed these in *C. elegans*. We show that these second site substitutions restore *in vivo *Aβtoxicity, further supporting the glycine zipper model.

**Conclusions:**

Our structure/function studies support the view that the glycine zipper motif present in the C-terminal portion of Aβ plays an important role in the formation of toxic Aβ oligomers. Compounds designed to interfere specifically with formation of the glycine zipper could have therapeutic potential.

## Background

Many studies support the view that accumulation of the β-amyloid peptide (Aβ) is central to Alzheimer's disease pathology [1]. Synthetic Aβ is toxic in both neuronal cell lines [2,3] and primary neurons [4], and Alzheimer's-like pathology has been observed in a range of transgenic models that accumulate Aβ [5-8]. Although numerous studies have implicated oligomeric Aβ as the toxic species [9-11], the structure of the key toxic Aβ species is unresolved, as is the toxic mechanism. As it has not been possible to obtain atomic structures for Aβ by X-ray crystallography, there is significant disagreement as to whether the toxic form of Aβ involves an α-helical or β-sheet structure.

**Figure 1:**
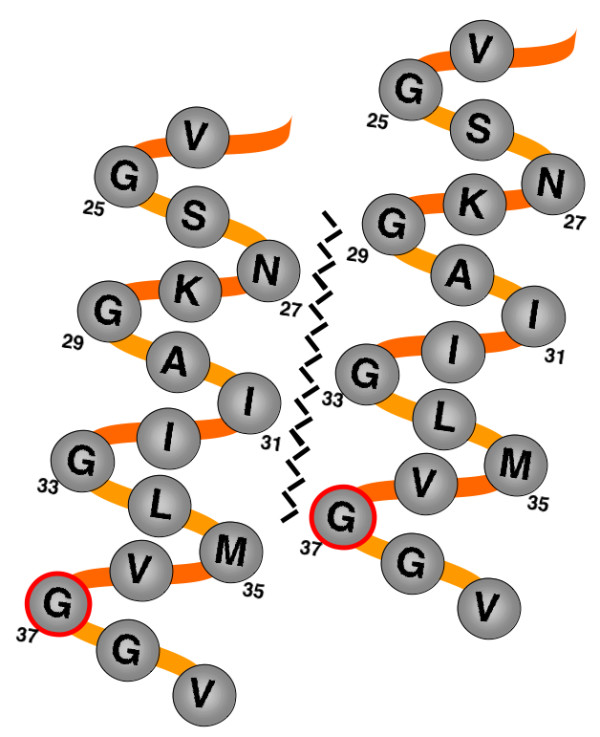
**Schematic model of hypothetical glycine zipper-mediated interaction between Aβα-helical regions**. Model of possible packing arrangement between C-terminal regions (residues 24-39) of neighboring Aβ molecules. The glycine zipper interface is represented by the jagged line; residue G37 is highlighted in red.

Synthetic Aβ preparations can convincingly form pores in synthetic membranes, leading to the proposal that *in vivo *Aβ toxicity results specifically from direct membrane damage [12]. Interestingly, the hydrophobic C-terminal region of Aβ (corresponding to the transmembrane portion of the amyloid precursor protein, APP) contains a Gly-XXX-Gly-XXX-Gly motif, termed a "glycine zipper" by Kim et al [13]. These researchers pointed out that this motif is present in the transmembrane domains of a number of bacterial channel proteins, and structural modeling of these channel proteins suggests that the glycine zipper motif can drive the packing of transmembrane α-helices. A schematic model showing the potential glycine zipper motif interface between α-helical regions of two Aβ peptides is shown in Figure 1. To support the idea that the glycine zipper motif of Aβ drives the formation of membrane pores, Kim et al demonstrated that Gly-to-Leu substitutions in this motif (particularly the G37L substitution) could block Aβ pore formation in synthetic membranes and reduce Aβ toxicity in cultured Neuro 2a neuroblastoma cells. However, these studies did not directly demonstrate that the G37L substitution disrupted the formation of Aβ oligomers, or that oligomer disruption was the basis for the reduced toxicity in cell culture.

Hung et al [14] compared the toxicity of synthetic Aβ 1-42 containing G25L, G29L, G33L or G37L substitutions (generally termed GSL peptides) to wild type Aβ in primary mouse cortical neurons. These substitutions were all found to be less toxic than wild type Aβ, with the G33L and G37L substitutions having the greatest reduction in toxicity. The reduced toxicity of these GSL peptides was correlated with reduced formation of smaller Aβ oligomers (dimers, trimers, tetramers, and pentamers) *in vitro*. These researchers also found that the GSL peptides had increased rates of fibril formation using an *in vitro *Thioflavin T assay. Harmeier et al [15] examined the role of G29 and G33 in Aβ 1-42 toxicity and oligomerization, and found that substitutions at G33 (G33A or G33I) dramatically reduced Aβ toxicity in neuroblastoma cells and biased *in vitro *oligomerization towards higher molecular weight forms. These researchers also demonstrated that unlike wild type Aβ, G33-substituted Aβ did not inhibit hippocampal LTP or disrupt eye formation in a Drosophila transgenic Aβ expression model.

In theory, the relevance of the glycine zipper to Aβ toxicity could be explored in transgenic mouse models expressing Amyloid Precursor Protein (APP) that contains substitutions in the glycine zipper motif. However, this motif has been demonstrated to play a role in the dimerization of APP, and substitutions in the motif alter APP proteolytic processing and Aβ production [16,17], thus confounding any interpretations of this hypothetical model. We have therefore employed a transgenic *C. elegans *model [18] that directly expresses Aβ42 to assay effects of glycine zipper substitutions, particularly G37L, on toxicity and amyloid formation, neither of which has been previously tested *in vivo*. To address the important issue of whether the correlations between the effects of glycine zipper substitutions on oligomerization and toxicity are causally linked, we used multiple cell models to ask whether G37L substitutions are actually anti-toxic, a prediction of the toxic oligomer model. Finally, we used second site substitutions in Aβ G37L to test rigorously structural predictions of the glycine zipper model *in vivo*.

## Results

### Aβ42 G37L is less toxic than wild type Aβ *in vivo*

**Figure 2:**
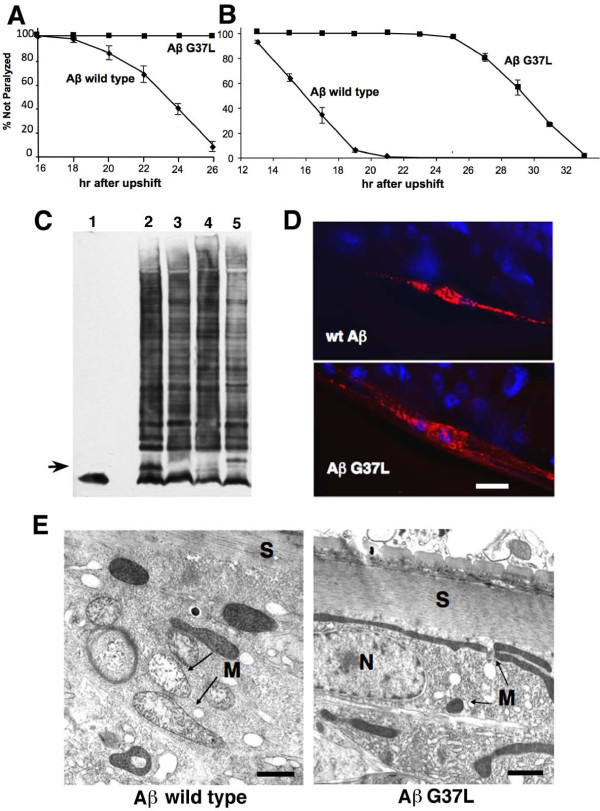
**Reduced toxicity of Aβ G37L expressed in *C. elegans***. **A**. Representative plot of paralysis onset in transgenic worms induced to express Aβ wild type (strain CL4176) or Aβ G37L (strain CL3523). In both of these strains the transgenes are marked with the dominant Roller mutation *rol-6(su1006)*. **B**. Paralysis plot for an independent pair of transgenic strains induced to express Aβ wild type (CL2659) or Aβ G37L (CL2621). In both of these strains the transgenes are marked with the intestinal GFP marker P*mtl-2*::GFP. **C**. Representative immunoblot of worm protein extracts (20 μg/lane) probed with anti-Aβ monoclonal antibody 6E10. **Lane 1**, 25 ng synthetic Aβ1-42; **lane 2**, CL4176 (Aβ wild type); **lane 3**, CL2659 (Aβ wild type); **lane 4**, CL3523 (Aβ G37L); **lane 5**, CL2621 (Aβ G37L). Note that although the overall patterns of oligomeric Aβ bands were similar in strains expressing Aβ wild type or Aβ G37L, differences did appear in the region of dimeric Aβ (arrow). **D**. Immunostaining with mAb 6E10 of transgenic worms expressing Aβ wild type (upper panel) or Aβ G37L (lower panel). Nuclei stained with DAPI (blue), 6E10 immunoreactivity in red. Size bar = 10 μm. **E**. Electron micrographs of sections of transgenic worms fixed by high pressure freezing. Left panel, section through body wall muscle of worm expressing Aβ wild type (CL4176). Note abnormal "light" mitochondria (arrows). Right panel, equivalent section through body wall of transgenic worm expressing Aβ G37L (CL3523). Note normal dark mitochondria (arrows). S = sarcomeres, N = nucleus, M = mitochondria. Size bar = 1 μm.

We have previously described and characterized a transgenic *C. elegans *model in which induction of human Aβ42 expression leads to intracellular accumulation of Aβ in body wall muscle cells, resulting in a highly reproducible paralysis phenotype [5,18]. To determine if a G37L substitution in Aβ would alter toxicity in this model, we generated transgenic strains (chromosomally-integrated transgenic arrays) with inducible muscle-specific expression of Aβ G37L, using either a dominant morphological marker [pRF4, *rol-6*(*su1006*), dominant Roller phenotype] or GFP expression [pCL26, P*mtl-2*::GFP, constitutive intestinal GFP] as co-transformation markers. We compared the paralysis kinetics of two independent strains [CL3523 (P*myo-3*::Aβ G37L minigene + pRF4) and CL2621 (P*myo-3*::Aβ G37L minigene + pCL26)] to paired control transgenic strains expressing equivalent levels of wild type Aβ (CL4176 and CL2659, respectively). (See Additional file 1 Table S1 for all transgenic *C. elegans *strains used in this study.) As shown in Figure 2 (panels A and B), both Aβ G37L strains had dramatically reduced rates of paralysis in comparison to the control strains. Despite the reduced paralysis rates, these strains had similar levels of Aβ expression (Figure 2C), and the distribution of immunoreactive intracellular Aβ deposits was not changed in the Aβ G37L strains (Figure 2D). We did note a variable pattern of immunoreactive bands between strains (arrow, Figure 2C), but this did not consistently correlate with genotypes or phenotypes. In addition, a significant ultrastructural difference was observed between muscle cells expressing wild type and G37L variant Aβ: abnormal mitochondria with reduced electron density ("light" mitochondria) were observed in the large majority (> 85%) of body wall muscle cells expressing wild type Aβ while these were exceedingly rare in worms expressing Aβ G37L (Figure 2E, 1/71 muscles scored, 9 worms sectioned).

**Figure 3:**
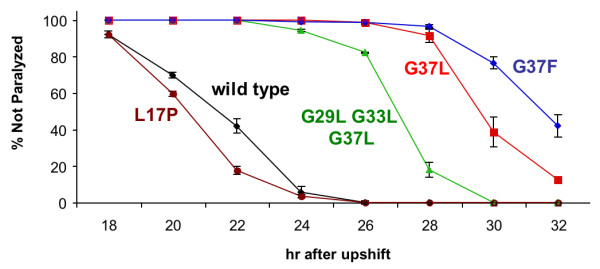
**Relative paralysis rates of worms expressing wild type or variant Aβ**. Representative plot of transgenic worms induced to express Aβ. Strains used were: CL2654 (Aβ L17P), CL2659 (Aβ wild type), CL2666 (Aβ G29L G33L G37L), CL2621 (Aβ G37L) and CL2716 (Aβ G37F).

The reduced toxicity of Aβ G37L in the transgenic *C. elegans *model could theoretically be due to the introduction of a leucine residue rather than removal of G37. We therefore constructed a transgenic line expressing Aβ G37F, and found that this substitution also dramatically reduced Aβ toxicity in the *C. elegans *model (Figure 3). An alternative interpretation for the reduced toxicity of the G37L substitution is that the glycine-to-leucine substitution simply increases the hydrophobicity of Aβ. However, we observed that a transgenic strain expressing the Aβ triple glycine zipper substitution (G29L G33L G37L) was actually slightly more toxic than Aβ G37L. While the model of Kim et al [13] predicts that the reduced toxicity of glycine zipper substitutions results from an interruption of α-helix packing, these substitutions could also conceivably interfere with β-sheet formation. We have previously demonstrated that a leucine-to-proline substitution at Aβ position 17 (L17P), which is strongly predicted to interfere with β-sheet formation by the central hydrophobic core of Aβ, blocks *in vivo *amyloid formation [19]. We therefore compared the toxicity of induced Aβ L17P to Aβ wild type and Aβ G37L, and observed that the toxicity of the L17P variant was comparable to that of wild type Aβ Figure 3. While this result does not rule out the possibility that the G37L substitution disrupts β-sheet formation, it does show that interfering with the ability of Aβ to form β-sheet structure does not by itself reduce Aβ toxicity in this model.

**Figure 4:**
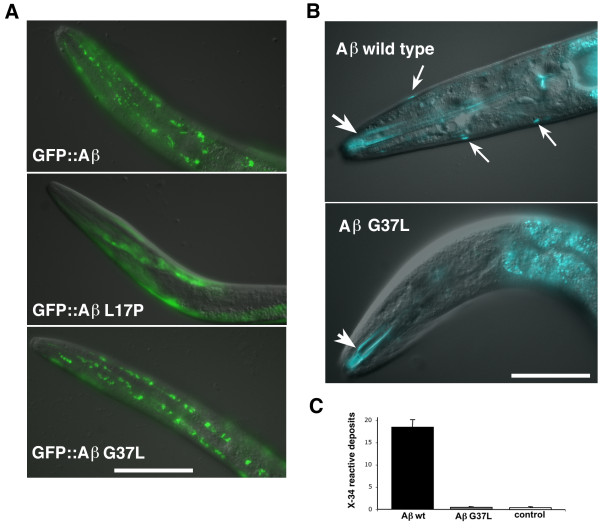
**Effect of G37L substitution on Aβ mediated aggregation and β-amyloid formation**. **A**. Digital overlay of differential interference contrast (DIC) and epifluorescence images of live transgenic worms expressing GFP::Aβ fusion proteins. Top panel, third larval stage worm expressing GFP::Aβwild type fusion protein (strain CL1332). Note GFP inclusions in body wall muscle. Middle panel, third larval stage worm expressing GFP::Aβ L17P fusion protein (strain CL1364). Note diffuse GFP in body wall muscle. Lower panel, third larval stage worm expressing GFP::AβG37L fusion protein. Note similar GFP inclusions as observed in worms expressing GFP::Aβ wild type (top panel). Size bar = 100 μm. **B**. Amyloid formation in transgenic worms with constitutive muscle expression of Aβwild type or G37L. DIC/epifluorescence images of live adult transgenic worms stained with amyloid-specific dye X-34 (33). Top panel, CL2006 (wild type Aβexpression). Note lateral amyloid deposits (small arrows) in body wall muscle and non-specific staining of the buccal cavity (large arrow). Lower panel, CL2564 (AβG37L). Muscle X-34 deposits are undetectable, although staining is still seen in the buccal cavity (large arrow). Size bar = 100 μm. **C**. Quantification of X-34 deposits in CL2006 (Aβwild type), CL2564 (AβG37L), and CL802 (control non-transgenic) worms. Error bars = S.E.M.

We have previously shown that the Aβ sequence can act as a modular aggregation domain, such that the C-terminal addition of Aβ 3-42 to GFP converts this normally soluble protein to a strongly aggregating one [20]. Introduction of an L17P substitution into the Aβ sequence reverses this aggregation capacity, thus GFP::Aβ L17P is soluble when expressed in *C. elegans *or hippocampal neurons. To test if the G37L substitution reduced Aβ toxicity by similarly blocking Aβ aggregation, we examined the distribution of GFP::Aβ G37L expressed in *C. elegans *muscle. As shown in Figure 4A, GFP::Aβ G37L readily formed inclusions in *C. elegans*, demonstrating that the G37L substitution does not interfere with Aβ-driven aggregation *in vivo*.

Constitutive expression of wild type Aβ in *C. elegans *body wall muscle leads to the formation of intracellular amyloid deposits detectable with amyloid dyes Thioflavin S [5,19] or X-34 [21]. We generated worms with constitutive Aβ G37L expression in muscle (P*unc-54*::Aβ G37L minigene) and found that this Aβ variant did not make detectable amyloid (Figure 4B, C). Thus, while the G37L substitution did not block general Aβ aggregation, it did prevent amyloid formation in this *in vivo *model.

### Synthetic Aβ G37L is anti-toxic in Neuro 2a cells

**Figure 5:**
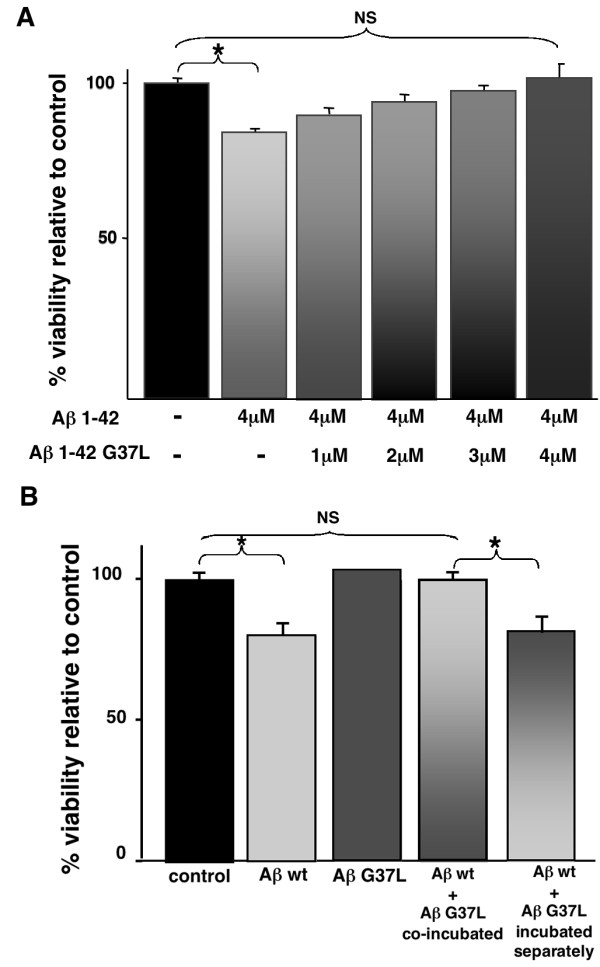
**Toxicity of Aβ wild type and Aβ G37L peptides on Neuro 2a cells**. Relative viability of Neuro 2a cells after treatment with Aβ wild type and Aβ G37L peptides for 24 hours, either singly or in various combinations. Viability was measured by the MTT assay and normalized to the vehicle-treated control cells. **A**. Neuro 2a cells were treated with 4 μM Aβ wild type that had been mixed with from 0-4 μM Aβ G37L for 20 min at RT before addition to the cells. The viability of each mixture was significantly different from that of Aβ wild type alone, and there was no significant difference in the viability of the vehicle control and an equimolar mixture of Aβ wild type and Aβ G37L (Student's t-test). Overall, Aβ G37L mitigated the toxicity of Aβ wild type in a linear dose dependent manner (R^2 ^= 0.98). **B**. Neuro 2a cells were treated as described above with 4 μM Aβ wild type and/or 4 μM Aβ G37L. Note that the ability of Aβ G37L to inhibit Aβ wild type toxicity is lost when these peptides are oligomerized separately before addition to the Neuro 2a cell culture. (* P < 0.05, NS = not significant).

To investigate whether Aβ G37L is anti-toxic, we first turned to a transformed cell culture model where Aβ exposure could be carefully controlled. Kim et al (13) reported that synthetic Aβ G37L was significantly less toxic to Neuro 2a cells than synthetic wild type Aβ. To determine if the presence of Aβ G37L could inhibit the toxicity of wild type Aβ to Neuro 2a cells, increasing amounts of Aβ 1-42 G37L were added to wild type Aβ 1-42 immediately after solubilization of these peptides. (Before solubilization, the peptides were rendered monomeric by pre-treatment with HFIP and subsequent desiccation.) As shown in Figure 5A, progressive addition of Aβ G37L led to a progressive reduction of wild type Aβ toxicity, such that at equal concentrations of wild type and G37L Aβ, loss of cell viability was equivalent to that of the vehicle control. These results could be due to a direct interaction between the wild type and variant peptide (e.g., Aβ G37L inhibiting the formation of a toxic oligomer by wild type Aβ), or an indirect effect (e.g., competition between the peptides for some toxic target). To distinguish between these possibilities, we compared the toxicity of equimolar mixtures prepared as described above to mixtures in which the individual peptides were solubilized and allowed to oligomerize separately for 20 min before addition to the Neuro 2a cells. As shown in Figure 5B, individual incubation of the peptides prevented Aβ G37L from interfering with wild type Aβ toxicity (P < 0.05, Student's T-test), consistent with the model that the Aβ G37L variant can directly interfere with the formation of toxic oligomers by wild type Aβ.

### Synthetic Aβ G37L is anti-toxic in mammalian hippocampal neurons

**Figure 6:**
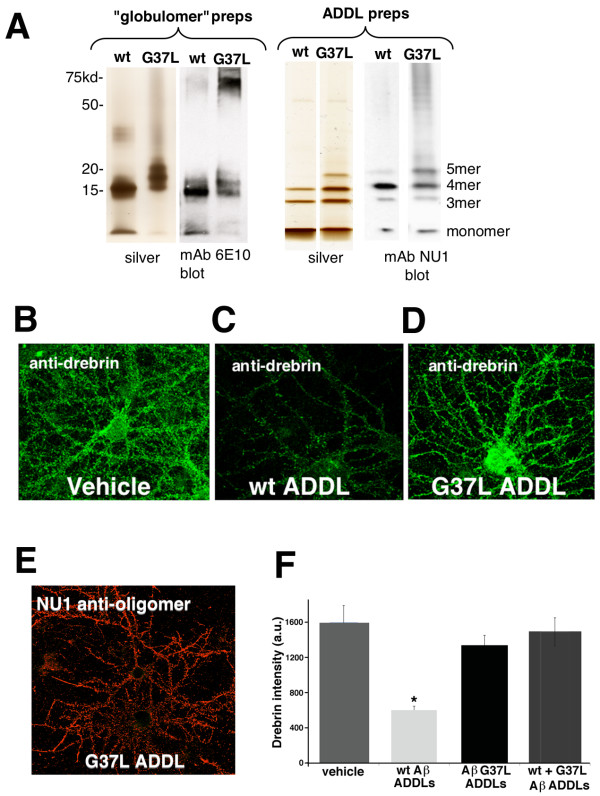
**Toxicity of Aβ wild type and Aβ G37L oligomers (ADDLs) in primary hippocampal cultures**. A. SDS-PAGE fractionation of oligomers formed from synthetic Aβ1-42 wild type or G37L. The left panel displays oligomers formed using the "globulomer" preparation of Barghorn et al [21]. The right panel displays oligomers formed using the "ADDL" preparation originally described by Lambert et al [24]. Monoclonal antibody 6E10 recognizes an epitope included in Aβresidues 16-24; mAb NU1 preferentially binds oligomeric Aβ. Note that for both oligomer preparations Aβ G37L tends to form higher molecular weight species. **B - D**. Representative epifluorescence images of anti-drebrin staining of embryonic rat hippocampal neurons treated after 21 days in culture for 24 hr with vehicle (**B**), 500 nM Aβ wild type ADDLs (**C**), or 500 nM Aβ G37L ADDLs (**D**). Note the strong reduction of drebrin staining is induced by exposure to wild type, but not G37L, ADDLs. **E**. Hippocampal neurons treated with Aβ G37 ADDLs, washed, fixed, and probed with anti-Aβ oligomer antibody NU1. Note robust binding of the Aβ G37L ADDLs to neurons. **F**. Quantification of drebrin immunoreactivity in hippocampal neurons exposed to Aβ wild type or G37L ADDLs. Exposure to Aβ wild type ADDLs significantly reduced drebrin immunoreactivity relative to vehicle-treated neurons (*P < 0.01), but no significant reduction was found for Aβ G37L ADDLs or ADDLs formed from a 1:1 mixture of Aβ wild type and G37L (Tukey Multiple Comparisons test).

In the experiment described above, we did not biochemically characterize the oligomerization of the Aβ peptides, and interpretation of the toxicity assay was limited by the use of immortal cultured cells. Aβ oligomers have been shown to be highly toxic when added to primary hippocampal neurons [22], so we attempted to replicate the Neuro 2a findings in a more disease-relevant primary neuronal culture system. We first examined the ability of synthetic Aβ 1-42 G37L to form oligomers *in vitro*, using two oligomerization protocols: the globulomer protocol of Barghorn et al [23] and the ADDL (Aβ Derived Diffusible Ligand) protocol of Lambert et al [24]. As shown in Figure 6A, synthetic Aβ G37L readily formed oligomers *in vitro*, and formed higher molecular weight species than wild type Aβ 1-42, as has been previously reported for glycine-substituted Aβ peptides (14,15). The toxicity of G37L ADDLs was assayed using primary hippocampal neurons as previously described [25,26]. Drebrin expression was used as a specific read-out of Aβ toxicity, as this is a critical dendritic spine protein, and synapses are key targets of ADDL toxicity [25]. Exposure of hippocampal neurons to ADDLs (500 nM) formed from wild type Aβ caused a significant reduction in drebrin detected by immunocytochemistry compared to vehicle-treated cells. A comparable reduction was not observed when neurons were exposed to ADDLs derived from Aβ G37L (Figure 6 B, C, D). The reduced toxicity of G37L ADDLs did not result from the inability of these oligomers to bind to hippocampal neurons, as immunostaining with an oligomer-specific antibody (NU1) revealed a robust association of these ADDLs with neurons (Figure 6E). The Aβ G37L peptide was also anti-toxic in this model, as mixing together wild type and G37L peptides resulted in non-toxic ADDLs (Figure 6F). However, unlike the situation in Neuro 2a cells, the Aβ G37L was anti-toxic even when pre-incubated with primary neurons before addition of wild type Aβ ADDLs (Additional File 1 Figure S1).

**Figure 7:**
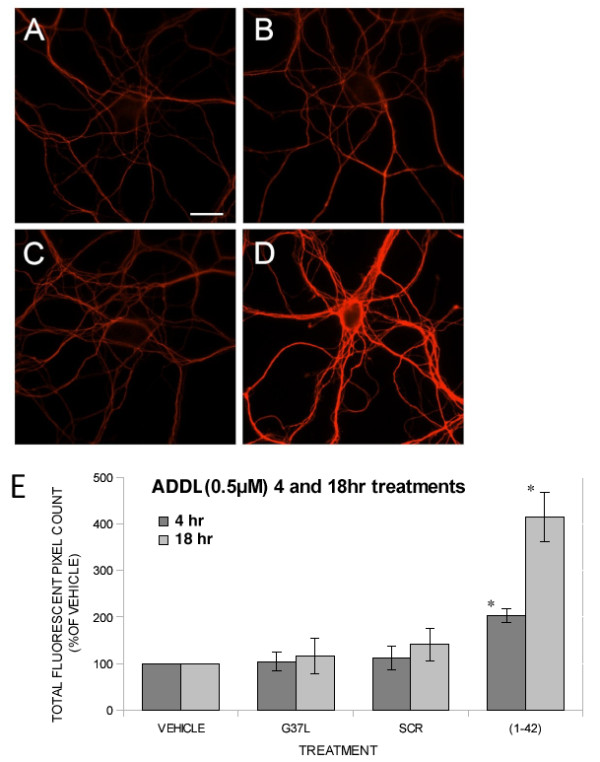
**Effect of Aβ wild type and G37L ADDL exposure on tau hyperphosphorylation**. A. - D. Representative epifluorescence images of cultured embryonic hippocampal neurons that were exposed for 18 hr to vehicle (**A**), 500 nM Aβ G37L ADDLs (**B**), 500 nM Aβ scrambled ADDLs (**C**), or 500 nM Aβ wild type ADDLs (**D**), then fixed and probed with anti-phospho tau monoclonal antibody PHF-1. Note strong increase in PHF-1 staining only in neurons treated with Aβ wild type ADDLs. **E**. Quantification of PHF-1 staining. Exposure to Aβ wild type produced significant increases in PHF-1 staining relative to control at 4 hr (204% ± 13; p = 0.0023; n = 5) and 18 hr (416% ± 48; p = 0.0041; n = 5); none of the other treatments significantly increased PHF-1 staining (Student's T-test, * P < 0.005).

Deposition of hyper-phosphorylated tau is an important component of Alzheimer's pathology, and Aβ ADDL oligomers have been shown to induce tau hyperphosphorylation in hippocampal neurons in culture [27]. We therefore also compared the abilities of wild type and G37L ADDLs to induce tau phosphorylation. We treated primary hippocampal neuronal cultures with ADDLs composed of wild type, G37L, or sequence-scrambled Aβ 1-42, and phospho-tau levels were quantified by immunofluorescence imaging after staining with PHF-1, an antibody that recognizes AD-related tau phosphorylation at residues Ser396 and Ser404. Vehicle-treated controls demonstrated low levels of phospho-tau (Figure 7A), similar to those observed in untreated neurons, whereas neurons treated with 0.5 mM wild type ADDLs had higher phospho-tau levels after 18 hours (Figure 7D). No difference was observed in neurons treated with either 0.5 mM scrambled ADDLs or G37L ADDLs (Figures 7B and 7C) at 18 hours compared to vehicle. Statistical analysis of quantitative immunofluorescence data revealed a twofold (P < 0.01) increase in tau phosphorylation in neurons treated with the wild type at 4 hours compared to vehicle, and a fourfold (P < 0.01) increase in tau phosphorylation at 18 hr (Figure 7E). Neither G37L nor scrambled ADDLs showed phospho-tau levels significantly different from vehicle-only treated neurons at either time (Figure 7E).

### Adenovirus-transfected Aβ G37L is anti-toxic in primary cortical neurons

**Figure 8:**
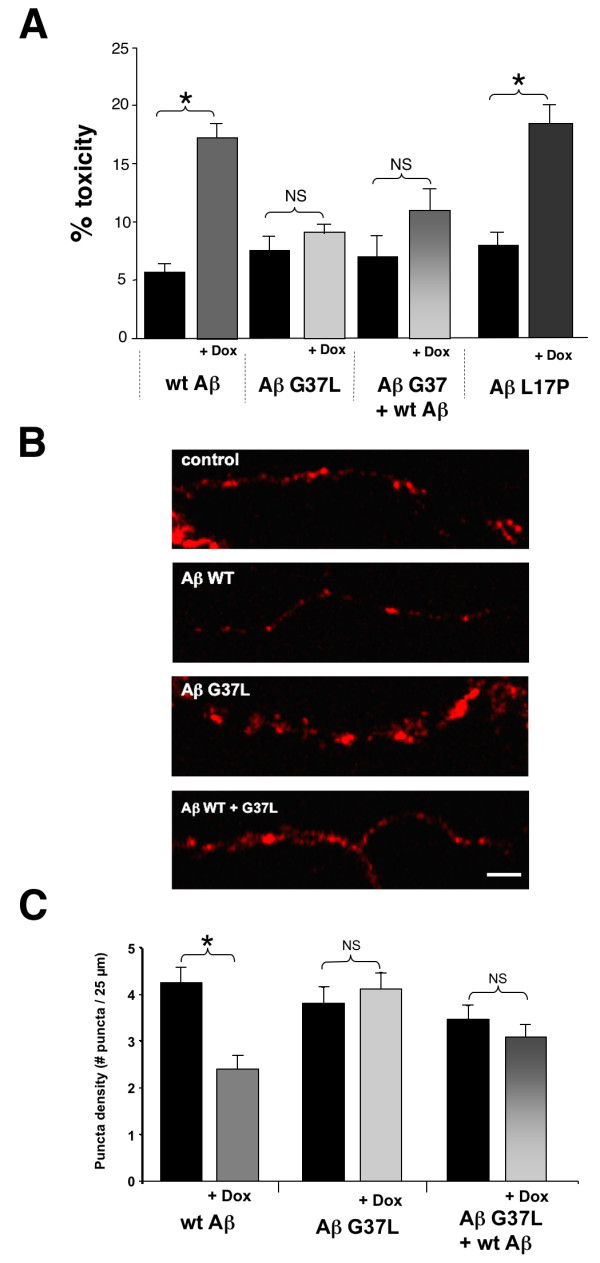
**Toxicity of wild type and variant Aβ in adenovirus transfection model**. Cultured rat cortical neurons were transfected with adenovirus engineered for doxycycline-inducible expression of wild type or variant Aβ, and toxicity was assayed by scoring of apoptotic nuclei or synaptophysin expression. **A**. Relative toxicity (pyknotic nuclei fraction) in neurons transfected with Aβ-expressing adenovirus with (+ Dox) and without induction. Transfection with adenovirus expressing Aβ wild type or Aβ L17P leads to significant toxicity after transgene induction (* P < 0.001), while transfection with adenovirus expressing Aβ G37L, or co-transfection with Aβ wild type and Aβ G37L, does not lead to significant toxicity after doxycycline induction. **B**. Representative processes containing synapses labeled with synaptophysin in Aβ wild type and Aβ G37L expressing neuronal cultures. Control panel refers to non-induced cultures. Scale bar, 5 μm. **C**. Analysis of puncta density (number of synapses per 25 μm of neurite) indicated fewer synapses in Aβ wild type expressing cells compared to non-induced controls. Intraneuronal Aβ G37L and co-expression of Aβ wild type and Aβ G37L resulted in no significant synapse alterations. Data from 3-5 different cultures from 3 independent isolations. n (neurites) = 154 Aβ wild type, 98 Aβ G37L, 118 Aβ wild type + G37L. (* = P < 0.001, NS = not significant, P > 0.1). Error bars represent SEM.

In the *C. elegans *model, cells were exposed to Aβ produced endogenously from an inducible transgene, rather than added exogenously as in the experiments described above. To replicate this pattern of Aβ expression in mammalian neurons, we turned to an inducible adenovirus transfection system [28,29]. This system employs engineered adenovirus that contain a signal peptide::Aβ 1-42 minigene (directly analogous to the Aβ minigene used in the worm model) under control of a doxycycline-inducible promoter. In this model, doxycycline induction after transfection of rat primary cortical neurons with a viral vector expressing wild type Aβ 1-42 leads to a significant increase in toxicity (measured by appearance of apoptotic neurons or reduced synaptophysin staining) compared to uninduced transfected neurons. As shown in Figure 8A, we found that neurons transfected with an adenovirus vector expressing Aβ G37L did not show a significant increase in apoptotic nuclei after doxycycline induction, consistent with the reduced toxicity of this Aβ variant. Co-transfection with both Aβ wild type and G37L-expressing adenovirus also failed to significantly increase the appearance of apoptotic nuclei relative to uninduced controls, again demonstrating the anti-toxic effects of Aβ G37L expression. Interestingly, Aβ L17P is as toxic as Aβ wild type in this model, consistent with the Aβ L17P toxicity observed in the transgenic *C. elegans *model.

As an alternative measure of Aβ toxicity, we also assayed the effect of transfected Aβ on synaptophysin, a well-studied presynaptic marker. As shown in Figure 8B, doxycycline induction of Aβ wild type significantly reduces immuno-detectable synaptophysin. However, induction of transfected Aβ G37L by itself or Aβ G37L + Aβ wild type does not result in statistically significant decreases in synaptophysin compared to uninduced controls (Figure 8C), consistent with the anti-toxic effects of Aβ G37L.

### Second site substitutions restore toxicity to Aβ G37L

**Figure 9:**
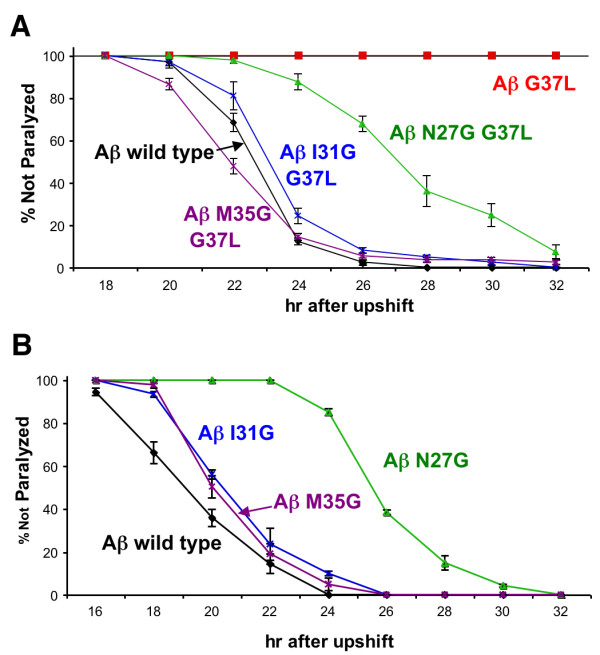
**Second site mutations partially restore Aβ G37L toxicity**. **A. **Paralysis plots of transgenic *C. elegans *expressing wild type, G37L, or double mutant Aβ. Note that the transgenic worms expressing the candidate compensatory mutations all paralyze faster than worms expressing Aβ G37L. **B**. Paralysis plot of transgenic *C. elegans *expressing single compensatory mutations. None of these single site mutations confer obvious hyper-toxicity.

A rigorous test of the glycine zipper model would be to determine if compensatory second site substitutions based on this model can restore toxicity to Aβ G37L. In models of glycine zipper interactions in bacterial channel proteins, the glycine-containing face of a membrane α-helix is opposed by the "backside" (i.e. opposite the glycine-containing face) of a neighboring α-helix [13]. If this model were applied to the formation of membrane-associated Aβ oligomers, Aβ G37 would be predicted to interact with the non-glycine face of an α-helix formed by a neighboring Aβ molecule. The specific residues interacting with Aβ G37 would therefore be those residing on the other side of the predicted Aβ α-helix, which are potentially N27, I31, or M35 (see Figure 1). If the disruption of the glycine zipper by the G37L substitution is due to the larger leucine residue sterically hindering α-helix packing, substituting a smaller amino acid opposite the leucine substitution could potentially compensate for the leucine substitution and thereby restore α-helix packing and Aβ toxicity. We therefore generated a set of transgenic worms expressing doubly-mutated Aβ: N27G G37L; I31G G37L; and M35G G37L. As shown in Figure 9A, all these second site substitutions increased the toxicity of Aβ G37L. Aβ with single N27G, I31G, or M35G substitutions were not more toxic than Aβ wild type (Figure 9B).

## Discussion

Although the β-amyloid peptide is toxic in a wide range of model systems, the specific toxic form of Aβ has not been convincingly identified, nor is it clear if the same form of Aβ is toxic in different model systems. Numerous studies have pointed to soluble, oligomeric Aβ as being the key toxic species, but there is no general agreement about the subunit composition or quaternary structure of this toxic oligomer. Defining the toxic Aβ species is difficult because while the oligomeric nature of Aβ can be assayed before it is added to cells (e.g., size exclusion profiling of ADDLs prepared from synthetic Aβ) or after Aβ is expressed (e.g., immunoblotting protein preparations from transgenic Aβ-expressing animals), capturing the toxic species "in action" has not been feasible. Direct biochemical approaches may also be problematic if the key Aβ toxic species is unstable and/or a minor component of total Aβ. To avoid these problems we have assayed the toxicity, *in vitro *and *in vivo*, of a set of Aβ variants designed to test a specific model of the toxic Aβ species. Our results demonstrate a critical role for C-terminal glycine residues in the formation of toxic Aβ oligomers, and thereby serve to significantly constrain structural models for these toxic oligomers.

We find that Aβ G37L is less toxic than Aβ wild type both *in vivo *and in primary neuronal culture. Our results are complementary to those of Harmeier et al [15], who showed that G33A or G33I substitutions also reduce Aβ toxicity *in vitro *and *in vivo*. (These authors did not report testing G37 substitutions.) These combined studies strongly argue that it is the disruption of the glycine zipper itself, rather than the alteration of any specific residue, that reduces Aβ toxicity.

How does Aβ assemble into the toxic oligomeric species? A large fraction of Aβ is membrane-associated both in the *C. elegans *model described here and in the AD brain (G. McColl and A. Bush, personal communication). We speculate that like a number of vertebrate antimicrobial peptides, Aβ exists in a β-sheet or random coil configuration when soluble extracellularly or in the cytoplasm, but converts to an α-helical structure upon membrane association. This conversion has been demonstrated by biophysical methods for plasticins [30,31] and bombinins [32], anti-microbial peptides that contain Gly-XXX-Gly-XXX-Gly motifs. Interestingly, Aβ has also been shown to have anti-microbial activity [33]. While x-ray crystal structures have not been determined for small anti-microbial peptides, the first atomic structure for an α-helical pore-forming toxin (ClyA) has recently been described [34]. We note that this study reveals a β-sheet "tongue" present in the soluble form of ClyA that converts to α-helical structure upon membrane insertion. This tongue region also contains a glycine zipper motif (...G^180^AAAGVVAG^188^...), and a glycine-to-valine substitution at position 180 has been shown to compromise hemolytic activity of this toxin [35]. Thus, although ClyA is significantly larger than Aβ, these structural data are consistent with our model of α-helix formation by the glycine zipper region of Aβ occurring upon membrane association.

Our observation that AβG37L does not form detectable amyloid *in vivo *differs from the results of Hung et al [14], who found that Aβ G37L had enhanced fibrilization *in vitro*. These results can be reconciled if there are significant differences in the amyloid seeding step [36] under *in vivo *and *in vitro *conditions. It has been proposed that the formation of α-helical structure by membrane-associated Aβ drives the formation of oligomers and therefore a local increase in Aβ concentration, and this localized Aβ concentration catalyzes β-sheet formation and amyloid formation [37]. α-helical intermediates have been observed preceding amyloid formation by Aβ [38]. We suggest that in the *C. elegans *model, glycine zipper-driven oligomerization at membranes is required to reach the critical local Aβ concentration to initiate amyloid formation, and this oligomerization and subsequent amyloid formation is blocked by the G37L substitution. However, other interactions (perhaps influenced by overall peptide hydrophobicity) may drive the formation of initial amyloid seeds *in vitro*, and thus the glycine-substituted Aβ peptides have faster amyloid formation rates under these conditions.

While Aβ G37L appears anti-toxic in both Neuro-2a cells and primary hippocampal neurons, we did observe different co-incubation requirement for these two model systems: inhibition of wild type Aβ toxicity required immediate co-oligomerization with Aβ G37L in the Neuro-2a model but not in the primary neuronal culture model. One possible explanation for these differences is that Aβ oligomers show highly preferential binding to synaptic sites in primary neurons [25], suggesting there may be a limiting number of Aβ binding sites in these neurons. Consequently, unoligomerized Aβ G37L may not only counter the toxic oligomerization of wild type Aβ, but oligomerized Aβ G37L may also compete with wild type oligomers for binding to relevant sites on the neuronal plasma membrane. Competition for binding sites may not be relevant to Aβ toxicity in the undifferentiated Neuro-2a cells used in our study.

Our demonstration of anti-toxic effects of Aβ G37L in cell culture and primary neurons clearly supports the oligomer model for Aβ toxicity, although we cannot readily infer the subunit composition of these oligomers from our experiments. We have observed variable patterns of presumed Aβ oligomeric bands in strains expressing different Aβ variants, but we have been unable to associate a specific oligomeric band with toxicity. A caveat to this approach to interpreting immunoblots is that we do not know to what degree they capture the true distribution of Aβ oligomers *in vivo*. It has been reported that different biophysical forms of Aβ actually show similar oligomer banding patterns after SDS-PAGE [39].

Even though Kim et al have demonstrated that the G37L substitution blocks Aβ channel formation in synthetic membranes, and we have rigorously demonstrated that Aβ G37L has reduced toxicity in multiple models, at present we have no direct evidence that the reduced toxicity of Aβ G37L is due to effects on membrane conductance *per se*. We also note that Aβ need not form pores in order to disrupt membrane function; instead it could result from Aβ altering the local membrane environment such that the conductance of endogenous ion channels (e.g., the NMDA receptor) is altered. An appealing component of the membrane disruption hypothesis is that it explains why Aβ is toxic when both outside cells (e.g., hippocampal neurons exposed to ADDLs) and inside cells (e.g., intracellular accumulation in *C. elegans*): in either case, Aβ has access to the plasma membrane. In theory, intracellular Aβ could also damage organelle membranes, such as the ER or mitochondrial membranes. Aβ accumulation has often been associated with mitochondrial dysfunction [40-42], and we have observed mitochondria with abnormal ultrastructure specifically in *C. elegans *muscles accumulating Aβ (Figure 2). Given that we did not observe abnormal mitochondria in transgenic worms expressing Aβ G37L, we speculate that expression of wild type Aβ in this model may lead to disruption of mitochondrial membrane function.

Introduction of second site mutations into Aβ G37L resulted in enhanced toxicity. This compensatory interaction is most striking in the N27G G37L variant, which is clearly more toxic than either single substitution (see Additional file 1 Figure S2). However, interpretation of the enhanced toxicity in Aβ I31G G37L and Aβ M35G G37L relative to Aβ G37L is complicated by the observation that the single Aβ I31G and Aβ M35G strains produce reduced levels of Aβ relative to the control Aβ wild type strain but show comparable paralysis rates, suggesting these single substitutions may be more toxic than wild type Aβ. If this is the case, we cannot exclude the possibility that these second site mutations increase the toxicity of Aβ G37L by an additive rather than compensatory mechanism. If the correct interpretation is that these second site mutations all increase toxicity by restoring helix packing in the glycine zipper region, our results imply that there is no single packing arrangement involved in the formation of toxic Aβ oligomers, as all three of the tested second site substitutions enhanced the toxicity of Aβ G37L. An alternative explanation for the enhanced toxicity of the double mutant Aβ is that it is the total number of glycine residues in the C-terminal region of Aβ that is the key determinant of toxicity. Although we cannot exclude this possibility, it is difficult to develop a molecular model that would account for this explanation.

We did undertake multiple attempts to generate transgenic worms with a leucine substitution at the single glycine residue in Aβ that lies outside the glycine zipper region (G9), which we predicted would not reduce toxicity. Unfortunately, all of the chromosomally-integrated transgenic lines recovered in this series of experiments did not produce detectable Aβ, an experimental failure that did not occur for any of the other glycine substitutions we attempted. It has also been more difficult to recover transmitting lines expressing wild type Aβ, presumably due to selection against lines expressing this toxic peptide (even under non-induced conditions). Thus, while we do not have data for the G9L control, we speculate that this variant may be at least as toxic as wild type Aβ.

Although Aβ G37L appears to be significantly less toxic than wild type Aβ when expressed in *C. elegans*, it does not appear to be non-toxic, as transgenic worms expressing Aβ G37L still ultimately become paralyzed. A possible explanation for this observation is that there may be two components to Aβ toxicity in this *C. elegans *model: a specific toxicity due to activity of Aβ oligomers, and a more general toxicity due to accumulation of misfolded protein. There is strong evidence that accumulation of misfolded proteins in *C. elegans *muscle can lead to a disruption of cellular protein handling (proteostasis) [43,44], and expression of an aggregation-prone polyglutamine::YFP fusion protein in *C. elegans *muscle leads to reduced motility [45]. Aβ G37L and the other variants examined in this study all make immunoreactive cytoplasmic inclusions when expressed in *C. elegans *muscle, consistent with our transgenic inducible system generally producing Aβ levels that exceed cellular protein handling capacity.

Our studies predict that compounds that specifically interfere with Aβ glycine zipper formation will block Aβ toxicity, and therefore would be candidate therapeutics for Alzheimer's disease. Computational methods exist to design peptides that specifically disrupt helix-helix interactions in protein transmembrane regions [46,47]; in theory this approach can be used to target the Aβ glycine zipper. Extending this approach to develop practical therapeutic compounds for Alzheimer's disease will likely require identification of brain-accessible small molecules that specifically inhibit Aβ glycine zipper interactions. Our studies also suggest the possibility that there may exist in the human population alleles of APP that encode substitutions in the glycine zipper regions that protect against Alzheimer's disease.

## Methods

### Construction and characterization of transgenic C. elegans strains

Transgenic constructs designed to produce temperature-inducible expression of substituted Aβ in *C. elegans *body wall muscle were generated from expression construct pCL12 (*Pmyo-3*::signal peptide::Aβ 1-42::long 3' UTR) [18]. The inclusion of an abnormally long 3' untranslated region in this construct leads to transgene expression being regulated by the mRNA surveillance system. Introduction of pCL12-derived transgenes into a genetic background containing a temperature-sensitive mutation in an essential RNA surveillance gene [*smg-1*(*cc546*)] results in increased transgene expression at non-permissive temperatures. Transgenes expressing variant Aβ were typically generated by *in vitro *mutagenesis [Gene Tailor kit (Invitrogen) or Site Directed Mutagenesis Kit (Stratagene)] of the signal peptide::Aβ 1-42 minigene cassette in a donor plasmid. The mutated Aβ minigene was then used to replace the wild type Aβ minigene sequence in pCL12 by restriction enzyme cleavage (Nhe I/Sac I) and religation. A transgene with muscle expression of GFP::Aβ G37L was similarly constructed by *in vitro *mutagenesis of pCL111 (P*myo-3*::GFP::Aβ 3-42). All plasmid constructs were confirmed by DNA sequencing.

*C. elegans *strains containing multicopy, chromosomally-integrated transgenes were constructed as previously described [18]. Briefly, Aβ expression plasmids (100 ng/μl final concentration) were co-microinjected with marker plasmids [100 ng/μl final concentration of either pRF4 expressing dominant Roller morphological marker *rol-6 *(*su1006*) or pCL26 expressing constitutive intestinal GFP (P*mtl-2*::GFP)] into *smg-1*(*cc546*) worms. Transmitting strains were initially recovered containing unstable extrachromosomal transgenes, and then strains were derived containing fully stable chromosomally-integrated transgenes by irradiation and subsequent selection (5). PCR and subsequent DNA sequencing was performed to check the Aβ genotype of transgenic strains.

#### Paralysis assays

Quantification of paralysis rates for transgenic *C. elegans *strains was done as previously described [48,49]. Briefly, populations of third larval stage transgenic worms were upshifted from 16°C to 25°C and the fraction of paralyzed fourth larval stage worms was scored every 2 hr starting 12-18 hr after upshift (depending on transgenic strains assayed). Triplicate populations (total n > 100) were scored for each genotype in a paralysis assay, and each paralysis assay was repeated at least three times. Transgenic strains containing the *rol-6 *morphological marker (which affects overall worm movement) reproducibly showed slower absolute paralysis rates compared to transgenic strains expressing the same Aβ construct but a GFP transgenic marker. All strain paralysis comparisons were therefore made between transgenic strains using the same marker transgene.

#### Quantification of Aβ expression in transgenic C. elegans

To quantify Aβ accumulation in *C. elegans *(Supplemental Table 1), transgenic populations were harvested 24 hr after temperature upshift, and whole worm extracts were fractionated by SDS-PAGE as previously described [50]. After blotting, the fractionated proteins were probed with anti-Aβmonoclonal antibody 6E10 (Abcam, #Ab10146) at 0.7 μg/ml, and Aβ immunoreactivity was visualized using HRP-conjugated secondary antibodies (1:1000 dilution), ECL development (GE Osmonics), and digital image acquisition (Image Station 2000R, Kodak). Total Aβ immunoreactivity was quantified using Image J software to sum whole-lane immunoreactivity (i.e., all oligomeric and monomeric bands as illustrated in Figure 2C). Aβ immunoblot expression was normalized to either CSTF64 (a ubiquitous, highly-expressed splicing factor) immunoreactivity or total blotted proteins assayed by Ponceau S staining.

#### C. elegans electron microscopy

Staged populations of transgenic *C. elegans *were shifted as third larval stage worms from 16°C to 25°C for 24 hr, then immediately harvested and subjected to high pressure freeze fixation as previously described [51].

#### Culture and treatment of N2a cells

Neuro2a mouse neuroblastoma cells (N2a) were grown in a 1:1 mixture of OptiMEM-I and DMEM (containing GlutaMax and nonessential amino acids), supplemented with 10% FBS. The cells were plated in poly-L-lysine-coated 96 well plates at 10,000 cells/well in 100 μL medium. Two days later, the cells were treated with wild type (wt) and/or mutant (G37L) Aβ42 peptides for 24 hours, whereupon their toxicity was assessed as a decrease in the metabolic reduction of MTT (3-(4,5-Dimethylthiazol-2-yl)-2,5-diphenyltetrazolium bromide) to formazan. Briefly, 10 μl aliquots of MTT (5 mg/ml in sterile PBS) were added to each well of cells for 2-4 hr at 37°C, the reaction was stopped by adding 100 μl 20% SDS in 50% N,N-dimethylformamide to each well, the plates were incubated at 37°C overnight to dissolve the purple formazan, and the absorbance was read at 560 nm. Frozen, dried preparations of monomerized Aβ-wt and Aβ-G37L peptides were dissolved in DMSO at 1 mM, diluted to 100 μM with OptiMEM-I, and immediately combined with additional vehicle, and/or with each other, to form mixtures that contained one or both peptides at a concentration of 25 μM each. These were allowed to sit at room temperature for 20 min, before being added to the cells at a final concentration of 4 μM for each peptide. In this way, N2a cells were exposed to either 4 μM Aβ-wt, 4 μM Aβ-G37L, a combination of 4 μM Aβ-wt plus 4 μM Aβ-G37L that had been incubated together for 20 min, or a combination of 4 μM Aβ-wt plus 4 μM Aβ-G37L that had been incubated separately for 20 min before being added to the cells together. In a variation of this protocol, the amount of Aβ-G37L peptide that was pre-incubated with 25 μM Aβ-wt was varied from 0-25 μM to determine how much Aβ-G37L was needed to counteract the toxicity of Aβ-wt peptide (applied to N2a at 4 μM). The amount of DMSO in the medium was the same (0.4%) for all samples and controls. In each experiment 4-6 wells were analyzed per treatment, and each experiment was repeated at least 3 times.

#### Exposure of hippocampal neurons to ADDLs

*Preparation of oligomeric Aβ*. Aβ oligomers (ADDLs) were generated from synthetic Aβ essentially as previously described [24,25]. Synthetic Aβ peptide was obtained from the following sources: Aβ1-42 and Aβ 1-42 G37L from American Peptide Company Inc., Sunnyvale, CA; scrambled Aβ 1-42 and fluorescein amidite-tagged Aβ1-42 from AnaSpec, San Jose, CA; and additional Aβ 1-42 G37L from Biosynthesis, Lewisville, TX. Peptides were prepared as dried HFIP films, then dissolved in fresh, anhydrous DMSO (Sigma Hybri-Max D-2650) to make a ~5 mM solution then diluted to ~100 μM with cold F12 media without phenol red (Biosource) and aged 24 hr at 4°C. The samples were centrifuged at 14,000 g for 10 min at 4°C and the supernatants stored at 4°C. Protein concentration was determined by Coomassie Plus protein assay (Pierce) with a BSA standard.

##### Gel electrophoresis of oligomerized Aβ

Samples were diluted in F12 and mixed 1:1 with Laemmli sample buffer (Bio-Rad) and electrophoresed at 125 V on a 4-20% Tris-glycine gel (Novex, Invitrogen) with Tris-glycine-SDS buffer (Bio-Rad) at room temperature (RT) for 100 min. The gel (45 pmoles/lane) was silver stained with SilverXpress (Invitrogen). Alternatively, the gel (15 pmoles/lane) was electroblotted onto Hybond ECL nitrocellulose using 25 mM Tris, 192 mM glycine, 20% methanol, 0.02% SDS, pH 8.3 at 100 V for 1 hr at 4°C. The blot was blocked with 5% non-fat dry milk in TBS-T (0.1% Tween-20 in 20 mM Tris-HCl pH 7.5, 0.8% NaCl) for 45 min at RT, incubated with NU1 (1 mg/ml in milk/TBS-T) for 2 hr at RT and washed 3 times with TBS-T. The blot was incubated with HRP-linked anti-mouse IgG (1:40,000 in milk/TBS-T; Amersham) for 60 min at RT, washed 3 times with TBS-T, rinsed 3 times with dH_2_0, developed with SuperSignal West Femto Maximum Sensitivity substrate (Pierce) for 1 min and read on a Kodak Image Station.

##### Preparation of primary hippocampal cultures

Hippocampi from embryonic day 18 Sprague-Dawley rats were used for the preparation of primary hippocampal cultures. For the drebrin expression experiments, cultures were prepared as previously described by Gong et al [22], and cultured neurons were exposed to ADDLs at 21 D.I.V. For the tau phosphorylation experiments, the primary cultures were prepared as described by Decker et al [52], and the cultures were exposed to ADDLs at 9 D.I.V.

##### Detection of Aβ binding to hippocampal neurons

ADDLs bound to mature hippocampal neurons were detected using monoclonal antibody NU1 [53] or by preparing fluorescent ADDLs (FADDLs) using biotinylated Aβ and streptavidin-conjugated quantum dots [54].

#### Immunocytochemistry, image acquisition, and analysis for drebrin expression

The drebrin expression assay was performed as previously described [26]. Briefly, mature hippocampal cells at 21 DIV are treated with the fluorescently tagged ADDLs (FADDLs) for 24 hr. Treated cells were rinsed to remove unbound Aβ-assemblies and then fixed with 3.7% formaldehyde or 4% paraformaldehyde in Neurobasal media (1:1 volume) for 20 min followed by an additional 20 min undiluted fixative. Cells were rinsed extensively in PBS. Cover slips were incubated in blocking solution (10% normal goat serum in PBS without 0.1% Triton X-100) for 45 min at room temperature (RT). Antibody against drebrin (Stressgen, Victoria, British Columbia, Canada) was used to identify dendritic spines. It was diluted in blocking solution and incubated on the cells overnight at 4°C. After rinses with PBS with 1% NGS, cover slips were incubated with appropriate Alexa-Fluor-conjugated secondary antibodies (Invitrogen, Carlsbad, CA) diluted in PBS plus 1% NGS for 90 min at RT, rinsed extensively in PBS, and mounted with ProLong Anti-fade media (Invitrogen).

Aβ oligomer-bound neurons and dendritic branches were imaged using a Leica (Exton, PA) TCS SP2 laser confocal microscope with laser intensity and signal detection settings held constant to allow for quantitative comparison between experimental groups. Optical serial sections of 0.5 μm intervals were taken through the cells and reconstructed to generate a maximum intensity projection of *z*-stack images from individual cells and dendritic branches. After setting the intensity threshold, dendritic segments of determined length were used to determine drebrin integrated fluorescence intensity. Spine Quantification was done using MetaMorph software version 6.3 (Universal Imaging Corporation, Downingtown, PA). All data are depicted as mean+/-SEM and were analyzed by GraphPad Prism4 software (San Diego, CA) for statistical significance using one-way ANOVA and, if overall *p *< 0.05, followed by a multiple group comparison *post hoc *test (Tukey's).

#### Immunocytochemistry, image acquisition, and analysis for tau phosphorylation

Neurons were fixed with a solution of 4% paraformaldehyde/4% sucrose in PBS for 15 minutes, rinsed two times in PBS and permeabilized for 7 minutes with Triton X-100 (0.25%) in PBS. The permeabilization solution was removed by rinsing three times in PBS. To prevent non-specific binding of antibodies cells were incubated with a Fish Skin Gelatin blocking buffer (FSGB) containing Fish Skin Gelatin (0.5%) (Sigma-Aldrich) and Triton X-100 (0.1%) in PBS. Neurons were double immunolabeled by overnight incubation at 4°C with mouse anti-PHF-1 (Ser396/404) (1:300) (provided by D. Volcado, Simon Fraser University, Burnaby, Canada) and rabbit anti-MAP2 (1:500) (Millipore, Temecula, CA) primary antibodies, diluted in FSGB. The cells were rinsed 3 times in PBS and incubated for 2 hours at 37°C with Cy-3 conjugated donkey anti-mouse IgG (1:500) (Jackson ImmunoResearch Laboratories Inc., West Grove, PA) and Alexa Fluor 488 goat anti-rabbit IgG (1:500) (Invitrogen) secondary antibodies, diluted in FSGB. Cells were rinsed 3 times with PBS and twice with Milli-Q water to remove buffer salts. Cover slips were mounted with Hoechst 33258 (10 μg/ml) (Sigma-Aldrich) in Elvanol.

Fluorescence imaging was carried out using a Leica DMI 6000B microscope (Leica Microsystems Inc., Richmond Hill, Canada) with a 63 × 1.4 plan apochromat objective (Leica Microsystems Inc.). Images were obtained with a cooled Orca-ER CCD camera (Hamamatsu Photonics, Bridgewater, NJ) controlled by MetaMorph (Molecular Devices, Sunnyvale, CA). Due to limitations in the digitizing range of the camera, pictures of treated specimens and controls sometimes had to be taken with different exposure times to avoid saturation of the maximum pixel value. The results were normalized for this difference during the quantitative analysis. Quantification of differences in tau phosphorylation was performed using Image J (NIH; Windows Version) essentially as described [27]. In brief, an appropriate threshold was applied to eliminate background signal before image analysis. Eighteen images per experimental condition were analyzed and at least two experiments using independent cultures were performed. Changes in tau phosphorylation were estimated by comparison of total fluorescent pixel counts and the results were combined for each condition. The total fluorescent pixel counts were normalized by the number of cell bodies in each image and by any difference in exposure times. Statistical testing was carried out using Microsoft Excel. Where applicable a student's unpaired t-test was performed. The mean value of 18 images from one experiment constituted one sample (n). An a level of 0.01 was used. Values for changes in tau phosphorylation are presented as mean ± standard error of the mean (SEM). Data for Aβ 1-42 oligomers (0.5 μM) at 4 and 18 hours were provided by H. Decker, Simon Fraser University, Burnaby, Canada.

#### Adenovirus transfections and experimental analysis - Antibodies

Anti-synaptophysin (Chemicon International, Temecula, CA) was used at a 1:250 dilution.

#### Adenoviral vector construction

The AdTet-On, AdTRE-LacZ, and AdTRE-Aβ42 viruses were previously described [28], G37L Aβ42 mutation was introduced by site-directed mutagenesis into the original pTRE-Aβ42 vector; the TRE cassette was subcloned into pShuttle vector, and recombination with pAdEasy-1 vector was achieved following manufacturer's instructions (Quantum Biotechnologies, Montreal, Quebec, Canada). Recombinant, E1-deleted replication-defective adenoviral constructs were produced [30]. Viral titer was determined by cell death assay.

#### Neuronal cultures

For rat primary cultures, cortices were dissected from embryonic day 18 Sprague Dawley rat brains (Charles River Laboratories, Wilmington, MA), and neurons were prepared as described previously [28]. Primary cortical cultures were infected 11-13 days after plating at final multiplicities of infection (m.o.i.) of 500. Equal amounts of Aβ42-WT and Aβ42-G37L viruses were used when added simultaneously. AdTet-On and AdTRE adenoviral vectors were added into culture for 18 h at a 1:5 ratio, and transgene expression was induced by 1 μg/ml doxycycline (Dox; Sigma) for 24 h. Efficiencies of transfection of > 70% were achieved.

#### Immunofluorescence

Cells were grown on poly-ornithine (3 μg/ml; Sigma) and laminin (5 μg/ml; Invitrogen, Carlsbad, CA) coated cover slips, washed once in PBS, and fixed in 3% paraformaldehyde in phosphate buffer as described previously [28]. The percentage of pyknotic nuclei was obtained by counting 20 random fields per sample at 40× as described [28], in each of 7-13 different cultures from 3 independent isolations. To analyze the effects of Aβ42 on synapses, images were collected in a Leica TCS SP5 Confocal Microscope (Leica Microsystems Inc., Bannockburn, IL) using a 63× lens, a standard pinhole of 1, optical intervals of 0.5 μm, 16-bit depth resolution, and with equal exposure times. MetaMorph software (Universal Imaging, West Chester, PA) was used for quantitative analysis. Threshold was set at least 2× above background levels. To analyze synaptic density (referred to as the number of synapses present in a given neurite segment) at least 5 random fields were imaged from each neuronal culture (3-5 different cultures from 3 independent isolations).

### Statistical analysis

Statistical significance was determined by the Student's two-tailed, unpaired t test, and a p value < 0.05 was considered indicative of a significant difference. All bar graphs are expressed as SEM.

## List of abbreviations

Aβ: β-amyloid peptide; N2a: Neuro2a; MTT: (3-(4,5-Dimethylthiazol-2-yl)-2,5-diphenyltetrazolium bromide): SEM: Standard Error of the Mean.

## Competing interests

The authors declare that they have no competing interests.

## Authors' contributions

*C. elegans *experiments were performed by VF, VD, CMR, PG, and CDL. Neuro 2a experiments were performed by GHS and ADDL-treated hippocampal were analyzed by PL. (oligomer binding and drebrin expression) and ND, EYF and MAS (tau phosphorylation). Adenovirus transfection of cortical neurons was performed by JM GHS, PL, MAS, JM and CDL prepared the manuscript. All authors have read and approved the final manuscript.

## Supplementary Material

Additional file 1 **"Aβ expression in transgenic strains and dose-response toxicity analysis for Aβ oligomers in hippocampal neurons**." This file contains measurements of relative Aβ expression and toxicity for all transgenic nematode strains used in this study (Table S1 and Figure S2) and quantification of drebrin expression in hippocampal neurons exposed to different levels of Aβ wild type or G37L oligomers. Click here for file
