# Correction: A glycine zipper motif mediates the formation of toxic beta-amyloid oligomers in vitro and in vivo

**DOI:** 10.1186/1750-1326-9-12

**Published:** 2014-03-25

**Authors:** Virginia Fonte, Vishantie Dostal, Christine M Roberts, Patrick Gonzales, Pascale N Lacor, Pauline T Velasco, Jordi Magrane, Natalie Dingwell, Emily Y Fan, Michael A Silverman, Gretchen H Stein, Christopher D Link

**Affiliations:** 1 Institute for Behavioral Genetics, University of Colorado, Boulder, CO 80309, USA; 2 Department of Neurobiology and Physiology, Northwestern University, Evanston, IL 60208, USA; 3 Department of Neurology and Neuroscience, Weill Medical College of Cornell University, New York, NY 10065, USA; 4 Department of Biological Sciences, Simon Fraser University, Burnaby, BC V5A 1S6, Canada; 5 Department of Molecular, Cellular and Developmental Biology, University of Colorado, Boulder, CO 80309, USA; 6 Integrative Physiology, University of Colorado, Boulder, CO 80309, USA

## Correction

After publication of this work [1], we noted that we inadvertently failed to include the complete list of all co-authors. The full list of authors has now been added and the Authors’ contributions section has been modified accordingly.

## Authors’ contributions

*C. elegans* experiments were performed by VF, VD, CMR, PG, and CDL. Neuro 2a experiments were performed by GHS. Characterization of ADDL preps was performed by PTV. ADDL-binding and toxicity assays in hippocampal cultures were performed and analyzed by PNL, and Tau phosphorylation analysis done by ND, EYF and MAS. Adenovirus transfection of cortical neurons was performed by JM. GHS, PNL, MAS, JM and CDL prepared the manuscript. All authors have read and approved the final manuscript.
