# Supplementary material for: A glycine zipper motif mediates the formation of toxic β-amyloid oligomers in vitro and in vivo
Source: Mol Neurodegener. 2011 Aug 23;6:61. doi: 10.1186/1750-1326-6-61 (PMC3178497; doi:10.1186/1750-1326-6-61)
Supplement: Additional file 1 — "Aβ expression in transgenic strains and dose-response toxicity analysis for Aβ oligomers in hippocampal neurons." This file contains measurements of relative Aβ expression and toxicity for all transgenic nematode strains used in this study (Table S1 and Figure S2) and quantification of drebrin expression in hippocampal neurons exposed to different levels of Aβ wild type or G37L oligomers. [file 1750-1326-6-61-S1.DOC]

| Strain (Rol transgene marker) | Aβ_42_ variant expressed | relative Aβ expression | P50 (hours) |
| --- | --- | --- | --- |
| CL4176 | wild type | 1.0 | 22.77 ± 0.07 |
| CL3523 | G37L | 1.28 ± 0.31 | >32 |
| CL3577 | N27G G37L | 1.45 ± 0.54 | 27.3 ± 0.25 |
| CL3578 | I31G G37L | 2.21 ± 0.45 | 23.43 ± 0.24 |
| CL4583 | M35G G37L | 2.45 ± 0.46 | 24.17 ± 0.52 |

| Strain (GFP transgene marker) | Aβ_42_ variant expressed | relative Aβ expression | P50 (hours) |
| --- | --- | --- | --- |
| CL2659 | wild type | 1.0 | 18.77 ±0.39 |
| CL2621 | G37L | 0.89 ± 0.2 | 29.9 ± 0.38 |
| CL2716 | G37F | 0.82 ± 0.16 | 29.93 ± 1.1 |
| CL2666 | G29L G33L G37L | 1.16 ± 0.2 | 28.13 ± 0.99 |
| CL2654 | L17P | 1.24 ± 0.2 | 19.93 ± 0.68 |
| CL3743 | N27G | 2.69 ± 0.16 | 25.3 ± 0.89 |
| CL2745 | I31G | 0.33 ± 0.58 | 19.0 ± 0.68 |
| CL3748 | M35G | 0.37 ± 0.22 | 21.7 ± 0.53 |

**Table S1**. **Relative Aβ expression of *transgenic C. elegans* strains.** Aβ expression levels were assayed by immunoblot and calculated from three biologically independent samples for each transgenic strain. Normalization of Aβ variant expression to Aβ wild type was accomplished by pairing the appropriate Aβ wild type sample (i.e., from strain CL4176 or CL2659) on the same immunoblot as the variant strain sample. Monoclonal antibody 6E10, used to detect Aβ in all experiments, recognizes an N-terminal epitope in Aβ and that is not affected in any of the variants. P50 refers to the time required for 50% of the population to become paralyzed (average of three biologically independent experiments.)

**
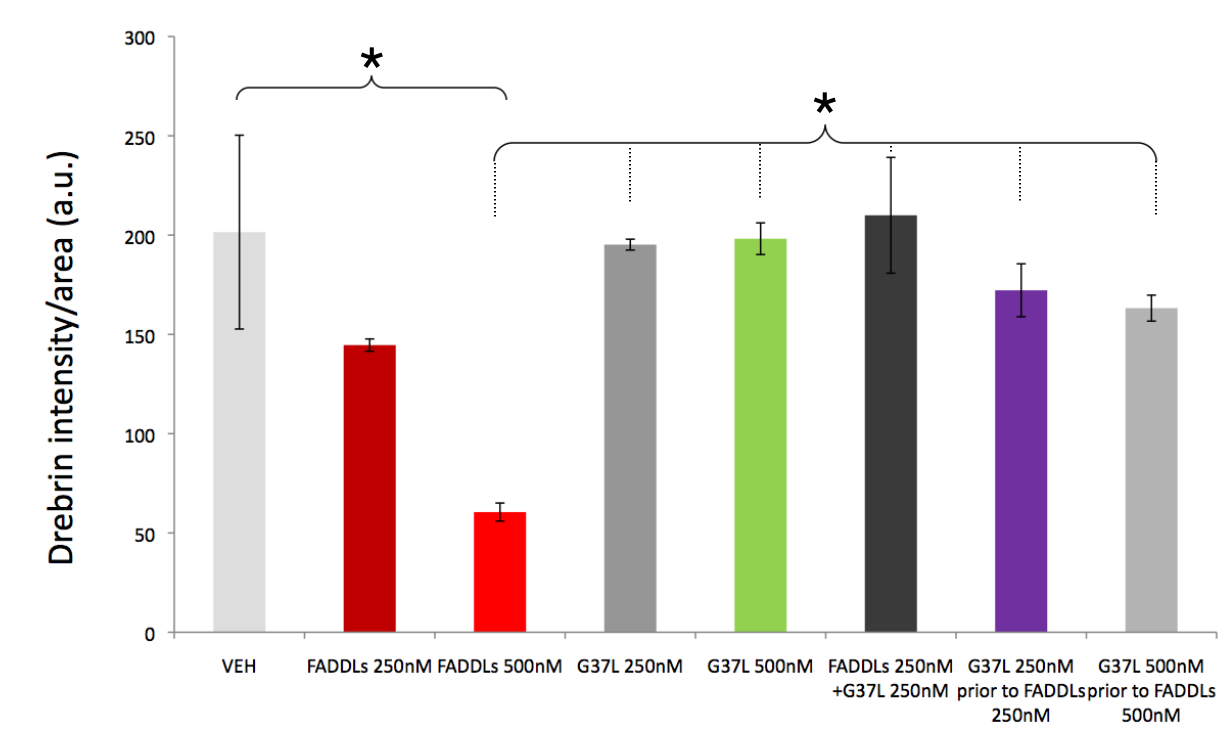
**

**Figure S1. Effects of dosage and preparation method on toxicity of Aβ wild type and A****β G37L oligomers (ADDLs) in primary hippocampal cultures.** Drebrin expression in cultured hippocampal cultures was quantified after exposure to fluorescently-tagged Aβ wild type oligomers (FADDLs), Aβ G37L oligomers, or mixtures of these peptides. The toxicity of FADDLs is similar to that of unlabeled Aβ wild type ADDLs (see Figure 6), and as observed for the unlabeled Aβ peptide, this toxicity is blocked by co-incubation of wild type and G37L peptides before addition to neurons (dark grey bar). In addition, prior exposure of neurons to G37L ADDLs blocks the toxicity of subsequently added wild type FADDLs, consistent with the possibility that G37L ADDLs compete with wild type FADDLs for membrane binding sites. 500nM FADDLs significantly decrease drebrin expression relative to vehicle treatment and all exposures with G37L ADDLs (*P <0.05, Tukey Multiple Comparison Test).

**
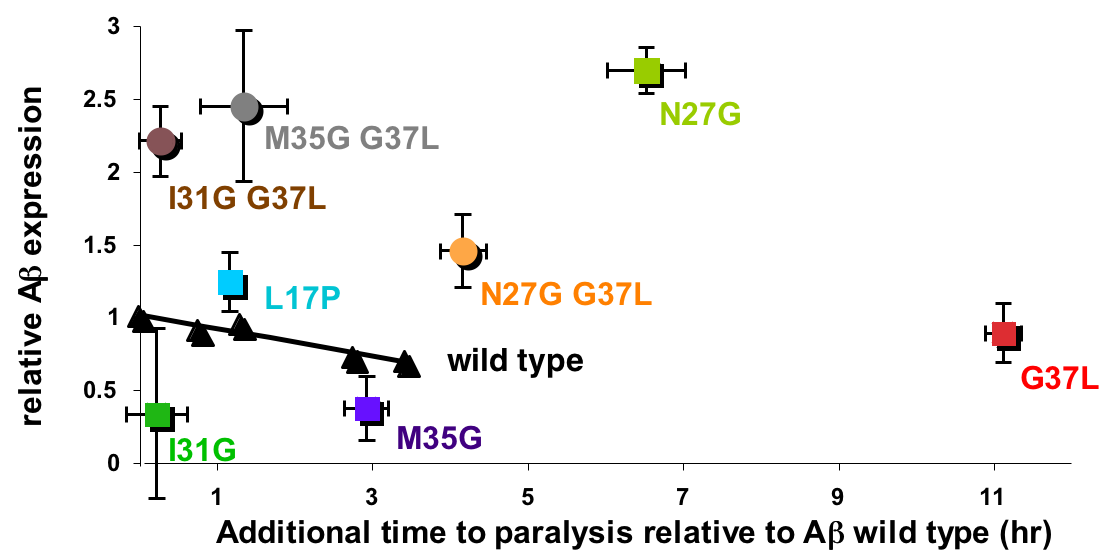
**

**Figure S2.** **Relative toxicity of variants is not a function of Aβ expression levels.** Data from Supplementary Table 1 are plotted along with a "standard curve" of toxicity for wild type Aβ, generated by decreasing Aβ expression in strain CL4176 by a dilution series of feeding RNAi against the Aβ transgene. Error bars indicate the SEM for triplicate measurements of expression (y axis) and additional time to paralysis relative to wild type (x axis). Decreasing wild type Aβ levels results in an approximately linear increase in paralysis times (black triangles and line). Although all variants shown had longer time-to-paralysis than the appropriate wild type Aβ control strain, the I31G and M35G strains had lower overall levels of Aβ expression, suggesting that these variants may be more toxic than Aβ wild type. Conversely, worms expressing Aβ N27G had a significantly longer time-to-paralysis than wild type Aβ, despite having significantly higher expression levels, indicating strongly reduced toxicity. Nevertheless, worms expressing Aβ N27G G37L show higher toxicity than either single variant, demonstrating these substitutions interact to increase toxicity.
